# Atlanta Academy of Medicine

**Published:** 1872-03

**Authors:** Jas. B. Baird

**Affiliations:** Reporter


### ATLANTA ACADEMY OF MEDICINE.

JAS. B. BAIRD, M.D., REPORTER.

DECEMBER 26, 1871.

Dr. Jas. F. Bozeman in the Chair.

Dr. W. F. Westmoreland reported that the case of the young man who had an epileptic form of convulsion, reported some weeks ago—to whom he had given large doses of bromide of potassium—continued to do well, having no return of his trouble. Has not taken the bromide for ten days, and feels no unpleasant effects from the discontinuance of its use. Has recently seen a lady who suffered greatly from neuralgia. Opiates only afforded temporary relief. Gave her heroic doses of the bromide of potassium, which afforded entire relief. Gave her four drachms the first day, two drachms the second day, and only one drachm on the third. Thinks a mistake is made in giving two small doses. Was induced to try large doses from reading a report of a case in which one ounce was given with no unfavorable result. These full doses are usually followed by certain nervous symptoms, which speedily pass off when the use of the bromide is suspended.

Has now on hand a case of diphtheria. Feels very uncertain as to the best mode of treatment. Would like to hear the opinion of the Academy on this subject. The case is that of a child aged four or five years; good constitution, florid complexion. Has constant fever; pulse not very frequent, sometimes even below the normal standard. The child is sometimes up, playing about the house, and in a few hours in bed again.

Dr. Bozeman has passed through two epidemics of this disease. Thinks no line of treatment should be invariably followed; the indications arising in individual cases should guide us. Does not remember to have seen a case under one year of age recover. Has generally found most benefit from sustaining plan of treatment—quinine, muriated tincture of iron, chlorate of potassa, anodynes, stimulants, and local applications to the throat. Generally prefers spirits of turpentine applied by means of a mop to the entire fauces; thinks it preferable to tincture of iodine or solution of nitrate of silver.

Dr. W. F. Westmoceland has an idea that the disease is constitutional; consequently, attaches little importance to topical applications. Is giving, in this case, quinine, paregoric, and concentrated solution chlorate of potassa in large doses, two drachms every three hours.

Dr. Bozeman has seen cases in which quinine seemed to produce an unfavorable excitement.

Dr. Cumming would rely principally on large doses of tincture chloride iron. For an adult give twenty drops every hour for four or six hours; and if the improvement is decided, gradually reduce the dose, but still keep up large doses, as you would in erysipelas. Thinks large doses necessary to bring out the good effects of the remedy. Ten drops three times a day is trifling with it. Has given half-drachm doses every hour till an effect was produced, and let down gradually.

Dr. Bozeman has seen it used in this way in yellow fever, with a view to its styptic action, but experience did not sustain the practice. Does not think the use of calomel allowable in diphtheria.

Dr. W. F. Westmoreland remarked a peculiarity in fatal cases of this disease. Remembers the first case he ever saw: a boy between four and six years of age; seemed not very sick—was up, playing about, one hour before death. Has met with several similar cases in the course of his practice.

Dr. Bozeman thought it a striking peculiarity. Physicians are frequently censured for under-estimating the gravity of the disease when this peculiarity is presented.

Dr. Raushenberg's experience is, that purgative doses of calomel are required in the beginning of the disease, as constipation is usually present. Passed through an epidemic in 1854 and 1855. Then treated a large number of cases. Gave large doses of calomel at the beginning, subsequently relying on tincture iron and quinine. Scarlatina prevailed at the same time. Rarely gives tincture of iron in less doses than twenty-five or thirty drops.

Dr. W. F. Westmoreland inquired why he preferred calomel to other cathartics.

Dr. Raushenberg replied, because he usually found a *bilious tongue*.

Dr. W. F. Westmoreland does not go so far as some gentlemen, and never look at the tongue, but he does not know what a *bilious tongue* is.

Dr. Raushenberg refers to the tongue as found in bilious fever—covered with a thick, dirty-yellow fur, brownish toward the middle, lighter on the edges.

Dr. W. F. Westmoreland.—Did you ever see a case of fever from any cause, even a cut finger, without a furred tongue? What occasions fur on the tongue?

Dr. Raushenberg would like to hear from Dr. Westmoreland on that subject.

Dr. W. F. Westmoreland thinks we attach too much importance to the appearance presented by the tongue. Some persons say the fur is produced by the passage of air over the tongue, incident to loss of teeth, etc. He does not go to that extreme, but does not rely upon it.

Dr. Cumming knows that when he had good teeth, his tongue was always furred; the roof of his mouth, palate and entire fauces were also covered, and could not be cleaned off. Has no doubt but that it is a wrong secretion of the tongue; takes a slight derangement to produce it. When it exists, the secreted mucous from the lining membrane of the mouth is much changed—very tenacious, sometimes yellow, even bitter. Thus some persons think it is bile eliminated by the mucous membrane instead of the liver.

Dr. W. F. Westmoreland.—Do you regard jaundice as an evidence of non-secretion on the part of the liver?

Dr. Cumming would not go so far as to admit that. Has suffered much from intermittent fever. The liver would become, at times, engorged and enlarged, projecting two fingerbreadths below the false ribs, accompanied by a sensation of oppression about the head, and a feeling that the whole system was poisoned. Thirty or forty grains of calomel, producing free alvine evacuations, was always followed by relief. Is inclined to think these evacuations consist largely of bile. When the liver works well, there is no fur on the tongue. In Amoy, China, has seen men in whom the tongue and lining membrane of the mouth presented the appearance of buckskin. Hard pulse, headache, pain in eyes, a sense of oppression in the epigastric region—the patient believing he would certainly die. Has given, in these cases, ipecac repeated, keeping up nausea and producing free vomiting to the amount of *three quarts of bile* in some cases, at the same time passing by the rectum bile of such acrid quality as to *burn the anus*. In four or six hours, the liver would draw up four fingers, be free from pain and tenderness, and the man would eat a hearty breakfast the next morning. Such cases are most frequently seen in the person of the first mate on rice vessels, their business requiring them to be in the hold of the ship, exposed to moist atmosphere at a high temperature.

Dr. Westmoreland must take issue, *in toto*, with Dr. Cumming. He thought the old idea, that bile might be secreted on the tongue and conjunctiva, had been exploded. If bile is secreted by any other organ than the liver, he does not know it. Thinks the cases referred to substantiate his position: a cathartic or an emetic is given, and the bile contained in the ducts passes out; relief due to cleansing the liver of contained bile. How many cases of jaundice are due to duodenitis! The bile is absorbed after it is secreted.

Dr. Cumming.—What has increased the size of the liver?

Dr. W. F. Westmoreland.—The three quarts of bile, you say, are poured out. It could not have been secreted in six hours. Do you contend that in jaundice the liver is doing its duty?

Dr. Cumming.—It may. But there are cases in which the liver is painful and tender on percussion; a congested, engorged condition obtains. Thinks a large part of the enlargement due to full bile ducts, and thus the function is interfered with. The kidneys fail, and uremia follows. So with the liver: poisoning follows a failure to perform its function. Relieve the congestion, and the man is well.

Dr. Westmoreland.—Why think the liver is not performing its function because painful? Is not a distended bladder painful?

Dr. Cumming.—Yes; but it is only a reservoir?

Dr. Westmoreland.—If a calculus blocks up the ureter, does the kidney fail to secrete?

Page(s) missing

Dr. Cumming.—No. When the liver is enlarged, something is wrong.

Dr. W. F. Westmoreland remarked that the remedies Dr. Cumming used proved his position. Ipecac produces relaxation and vomiting, and the ducts pour out the bile. If active congestion, approaching inflammation, existed, relief could not be obtained in six hours.

Dr. Cumming desired to impress that he did not say three quarts of bile were secreted in six hours, but had been poured out. The suffering is not caused by the enlargement of the liver, but from its failure to perform its function, caused by the congested state of the organ. When it is, *almost mechanically*, emptied, relief follows.

Dr. Westmoreland's great object is to defend the liver. Thinks it is a much-abused organ; gives people less trouble than many are inclined to believe.

Dr. Cumming.—Are you in the habit of examining livers?

Dr. Westmoreland.—Used to; rarely found them enlarged.

Dr. Cumming.—Possibly so in Atlanta; but enlargement is common in Savannah, Augusta, Columbus, and Amoy.

Dr. Westmoreland.—Why is the spleen not enlarged?

Dr. Cumming.—So it is when the patient is subjected to malarious influence.

Dr. Raushenberg thinks he has seen the *bilious tongue* so often, when no obstruction existed, that he cannot give up the idea. Thinks it due to deficient action of the liver.

Dr. Westmoreland.—How can the coloring matter be separated from the blood, except by the liver? In cirrhosis, there is no jaundice because there is no secretion. Has made *post-mortem* examinations of livers in this condition, in which not one-tenth part was capable of performing its function.

Dr. Cumming observed that the lungs and the kidneys were capable of doing more than was ordinarily required of them. Thinks the same is true of the liver.

Dr. Baird remarked that Dr. Cumming seemed to regard the bile as an *excretion* simply, whereas he believed it to be a compound fluid, composed of *excretory* and *secretory* matter—certain ingredients, as the biliverdin, the glyco-cholate and tauro-cholate of soda being formed in the gland for reabsorption for a special use in the economy, while the cholestein exists already in the blood, and is simply separated from the blood by the liver, to be thrown out of the system in the form of stercorine.

Dr. Bozeman.—Can the kidneys excrete bile?

Dr. Westmoreland.—Yes; they can *excrete* bile after it is reabsorbed by the blood.

Dr. Bozeman remarked that some cases of almost instantaneous jaundice, produced by a sudden mental emotion, militated against the theory of absorption.

Dr. Westmoreland replied that absorption takes place very rapidly. Coloring matter injected under the skin of a rabbit passes the round of the circulation and appears in the urine in a few seconds.

Dr. Bozeman was not sure that jaundice was produced by the absorption of bile after it is formed in the liver.

Dr. Westmoreland.—Then, where does the coloring matter of the bile come from?

Dr. Cumming desires the opinion of the Academy as to the best mode of breaking off the habit of opium-eating, and of avoiding the painful results that so often follow, as diarrhœa, neuralgic pains, etc.

Dr. Westmoreland thinks it depends upon the amount taken; if it is moderate, cut off the supply at once. A few years ago, saw a remarkable case. A gentleman assured him that he was in the habit of taking one drachm of morphine daily. He then had *delirium tremens* from morphine. His allowance was gradually decreased to five grains daily. He was unwilling to abandon the habit entirely, and it is usually a failure unless the patient gives his hearty coöperation in breaking off the habit. In some cases, where the quantity taken is large, he is in the habit of filling a bottle with laudanum, and when a dose of the laudanum is taken, its place is supplied by a like amount of water. Thus the laudanum is taken in a more and more diluted form, and soon none is taken at all. If it is taken in small quantities, and suddenly shut off, the patients suffer greatly for a few days; but let them suffer—it won't last long.

Dr. Bozeman has found iron and whisky the best substitute. He has not been able to get along without the alcoholic stimulant; after a while, can substitute assafœtida and valerian. Believes in sudden breaking off, but the patient must be sustained.

Dr. Westmoreland's views have recently changed in regard to stopping, suddenly, the use of stimulants. Not long ago, saw a sportsman, of whom it was said that he had not been sober for fifteen years. Drank three quarts of whisky the day before he saw him; drank one quart in the four hours immediately preceding his visit. The supply was cut short off, and he knows he drank no more for two months. He suffered a great deal for four days, but then began to take soup and eat regularly, and improved rapidly.

JANUARY 2, 1872.

The President, Dr. Logan, in the chair.

After an address of some length by Dr. A. Means, giving his peculiar views respecting electricity in its relation to life, its influence in health and disease, etc., Dr. W. F. Westmoreland was called on for his views in regard to this powerful agent. He responded by saying:

For a great while he had been impressed with the importance of electricity as a remedial agent, and more so, recently, since observing, in New York, the effect of electro-therapeutics—particularly as applied to surgical diseases. He believes it produces a decided effect upon malignant growths, as well as those of a non-malignant character. Has seen goitre cured by it in one or two sittings. Fatty and fibroid tumors are said to be cured by it.

Dr. J. Marion Sims and other gentlemen of New York apply it to *fibroids* of the uterus. The result, in many cases, is perfectly satisfactory. It is impossible, at present, to say what may be accomplished by it in the treatment of malignant diseases.

Dr. Frank H. Hamilton, of New York, insists that its application in cases of cancer relieves the peculiar cancerous pain for a certain length of time—for ten, fifteen or twenty days—and at the same time, in the majority of recent cases, where there is no loss of tissue, or where there is too great loss of vitality, allowing slough, it greatly diminishes the size of the tumor. In one case recently treated, the size of the tumor was diminished from one-third to one-half in ten days. How is this accomplished? By chemical changes in the growth itself. If ulceration exists, it is more perceptible, for the escape of gas may be seen and heard from the punctures produced by the needles. This effect follows the use of negative electricity. When the positive and negative poles are both introduced, destruction of the healthy as well as the diseased tissue is the result. The best results follow the use of the negative pole. The needle, attached to the negative pole, is introduced wherever a hardened point is found on the surface of the tumor; the electricity is applied for about fifteen or twenty minutes, and gas escapes on the withdrawal of the needle. Feels certain that the result is due to chemical changes. There is no inflammatory process. A few days suffices to effect a marked reduction in the size of the growth, and diminution in the amount of pain. Thinks that the day is not far distant when it will stand at the head of the list as a therapeutical agent, both in medicine and surgery, and believes that it will prove invaluable in the *neuroses*, etc. He is now using it in three cases, which he will describe, and report upon them as they progress.

Case No. I.—Returned cancer of the mammary gland. The breast was amputated about eighteen months ago. There is considerable loss of tissue; axillary glands enlarged; frequent hemorrhages, causing great debility. Application made.

Case No. II.—Returned cancer. Operated on last July; returned on the opposite side. As the lady did not bear chloroform well, the application was imperfectly made, ten or fifteen days since. Pain entirely relieved.

Case No. III.—A lady; cancer of the mammary gland of two years' standing. Glands in axilla enlarged, with shooting pains. After the first application, she experienced no pain for six or eight days. She returned on the eleventh day, to obtain relief from pain. The tumor was reduced from one-fourth to one-third in size, the hard, stony feeling gone, and the circulation in the skin much improved.

Dr. J. S. Wilson added, that there could be no doubt as to the diagnosis in these cases; they were undoubtedly malignant. He heard the gas escape, and discovered it by the odor. Believes a chemical decomposition is produced in the tumor.

January 29, 1872.

The President, Dr. Logan, presiding.

Dr. Cumming reported a case illustrating a point made in the report of the Section on Hygiene, recently.

Was called to see a lady suffering from slight dysentery; nearly the whole of the large intestine seemed to be involved. He gave ipecac in large doses. She was relieved for forty-eight hours. Much to his surprise, it then returned. The case was then more thoroughly investigated, and it was found that the whole economy was devoted to furnishing milk to her infant, which had made the mother almost anæmic. Her stomach became very irritable, so that the most simple article of food was rejected. He found that the child was drawing from the mother all the nourishment she received. Thus the child was in fine condition, while the mother was greatly reduced. Feared the necessity of separating them, but relief was obtained without a resort to this measure. Thinks that the woman lost ground during gestation, and since the birth of her child, most of her nutriment has gone to milk. She requires an abundant supply of good food, plenty of fresh air and sunshine, to enable her to go through the remaining ten months of lactation; the child is now only five months old. The vigor of a child defends it from disease. A strong child resists agencies that a feeble child would succumb to. Hygiene is more important than medicine, but it requires a certain amount of foresight and enthusiasm on the part of the physician to carry it out.

Dr. Bozeman inquired why Dr. Cumming gave ipecac, and in what doses was it given?

Dr. Cumming replied that he gave four doses, of ten grains each. In India, where acute dysentery is *the* disease, it is relied on to the exclusion of other remedies.

Dr. Bozeman.—Is aware that it has been used, but don't see the wisdom of giving it to a feeble woman. You certainly do not wish to excite the emunctory system. Put her to bed, give small doses of opium, and a better result will be obtained, and it will be got sooner. Does not think it will do to rely upon it to the extent that Dr. Cumming seems to do. Has seen good results follow the use of salts and laudanum, and calomel, and at other times they would all fail. So it is with ipecac.

Dr. Cumming.—Admits Dr. Bozeman's criticism of the treatment in this case. If he had known the exact condition of the person, he would have modified it somewhat. Confesses that he was deceived as to her condition by the fine appearance presented by the child; knows, now, that she was rendered anæmic by excessive lactation.

The President.—Is the final improvement due to medical treatment, or did it occur irrespective of it?

Dr. Cumming.—Not so much to medical as to hygienic treatment. She was put on chicken and beef tea and milk, but not in sufficient quantities to sustain her. Then put her on oyster soup and chicken tea. She is now eating solid food, and using claret.

Dr. Jno. Stainback Wilson.—Has now a case—a woman with a child nearly six months old. She suffers from profuse menstruation; this and lactation (not excessive) is breaking her down. Shall the child be weaned?

Dr. Bozeman inquired if it was menstruation or hemorrhage.

Dr. Wilson replied that it was genuine menstruation, but more abundant than before her pregnancy.

Dr. Cumming.—The woman cannot be a good nurse if she can supply only one-half the necessary amount of milk. May not the menstruation be due to a failure of the breasts to do their duty? Put her on tincture iron—forty drops daily—and strict hygienic treatment. Women may ovulate and lactate. After making trial of this treatment, it may be found necessary to separate them, and put the child on cow's milk, properly prepared.

Dr. Wilson.—She can use only one breast, and the child has flourished partly on a diet of cow's milk. Iron brings on the menstrual flow.

FEBRUARY 12, 1872.

The President, Dr. Logan, presiding.

Dr. Cumming reported a case of neuralgia recently successfully treated by him. He remarked that neuralgia is usually treated as neuralgia alone, without a due regard to the cause producing it. This case had been treated by a number of physicians as a common cold, relying on anodynes. He felt sure that it was occasioned by malaria, though the patient denied having ever lived in a malarious district. After carefully interrogating the patient, he found that he was engaged in a campaign on the coast of Virginia during the late war, and was there exposed to malarious influences, having had several attacks of intermittent fever at that time. The treatment consisted of twelve or fifteen grains of quinine daily. The neuralgia was entirely relieved. The patient is now taking arsenic.

Dr. J. G. Westmoreland inquired if Dr. Cumming regarded quinine as a remedy for malaria alone. He had used it in cases of face neuralgia (commonly called *sun-pains*) and other neuralgias, with perfect success. Malaria had nothing to do with the causation of some of these cases. Does not think that, because quinine cured, it was of malarial origin.

Dr. Cumming.—*Sun-pains* are not known out of a malarial region, whereas everybody knows what they are in malarious districts.

Dr. J. G. Westmoreland.—Has lived in a malarious region, and lived in Atlanta when there was no malaria here, and saw sun-pains in this city then.

Dr. Cumming rejoined that nobody was born in Atlanta until comparatively recently. The persons affected with sun-pains may have come from a malarial region, and brought malaria with them.

Dr. Bozeman.—Thinks quinine just as applicable in certain cases of muscular rheumatism as in neuralgia. Recently saw a young man with violent pain in both sides, near the spine, brought on by excessive use of dumb-bells. Five grains of quinine, three times during the day, relieved him in twelve hours.

Dr. W. S. Armstrong.—Was raised in a non-malarial district. Never saw a case of chills and fever until he had been engaged in the practice of medicine for more than two years. Sun-pains were common there.

Dr. Cumming.—Thinks a slight exposure to malarious influences will produce sun-pains; a large amount of the poison will produce chills and fever.

Dr. J. G. Westmoreland.—Does not think the use of quinine should be restricted to malarial diseases, but that its application should have a range that its therapeutical value entitles it to.

Dr. Raushenberg inquired if Dr. Cumming believed that all periodical attacks were due to malaria.

Dr. Cumming.—Was not prepared to generalize to that extent, but does not contend that quinine should be confined in its application to malarial diseases.

Dr. Jno. Stainback Wilson.—Uses quinine in a great variety of diseases. Thinks ten or twelve grains, and a warm foot-bath, the best treatment for a common cold in its incipiency; frequently aborts it. It equalizes the vascular and nervous action whenever there is a loss in balance of the circulation. Thinks it is valuable in tetanus, and that it is a great error to confine its use to cases of supposed malarial origin.

Dr. Ray reported a case of cerebro spinal meningitis—a negro boy, aged ten years. Head drawn back; delirium; jaws tightly closed; pulse quick. Treatment consisted of ten-grain doses of quinine, sinapisms to the spine, and calomel. The medicine had acted by the day following, but there was no improvement in the symptoms. Urine passed involuntarily. At the suggestion of Dr. W. F. Westmoreland, he was put on quininine grs. iij., calomel grs. j., every three hours. Purpura was a prominent symptom in this case.

Dr. W. F. Westmoreland stated that he had seen the patient, with Dr. Cumming, this evening, it being the second day of the attack. There is a marked improvement in his condition. Yesterday his pulse could not be counted; now it is one hundred and twenty a minute, and regular. The ecchymosed condition is disappearing. The spots were dark-colored yesterday; they are livid to-day.

Dr. Ray added that a blister was applied to the whole length of the spine on the second day.

Dr. W. F. Westmoreland.—Saw another case last Tuesday night—a young man aged seventeen years, well grown and athletic. Was attacked Monday. Purpura distinct. Was pulseless when he saw him. He died seven hours later.

Dr. Boring.—Saw a case, in consultation, on Thursday afternoon—a boy eleven years old. Friday morning, at six o'clock, he had a congestive chill. Saw him at half-past ten Friday morning again. Was prostrated; covered all over with purple blotches, a blue circle around both eyes; almost pulseless. Put him on quinine, two grains every two hours. Saturday morning, he was better; Saturday night, congestion occurred again. Spots on the surface disappeared; became blind in the right eye; left eye congested. A third physician was then called in. The two grains of quinine were increased to five grains every two hours. He died at ten o'clock Sunday night. Never saw the spots so well-marked as they were in this case.

Dr. W. F. Westmoreland remarked that the peculiarity of the case he reported consisted in its resemblance to cholera morbus—almost constant vomiting and purging.

Dr. Jno. G. Westmoreland reported a case seen at ten A.M. Sunday—a girl aged six years. The afternoon previous, she complained of headache; toward night, a fever set up, which continued, with delirium, vomiting and convulsions, during the night. She would drop to sleep, and wake up greatly frightened and agitated; head drawn back. Determined that active measures were necessary. Bled her four or five ounces. She became pale and quiet. Applied a blister to her spine, from her neck to her sacrum, and gave fifteeen grains of calomel. She remained quiet during the day; had no vomiting or convulsions. This morning she was rational, quiet; gives evidence of recovery. At the same time saw her sister, a girl aged thirteen years, with pneumonia, the right lung being extensively hepatized. Bled her, and gave veratrum and nauseants to allay the high fever and the cough. Better to-day.

Dr. Raushenberg.—Saturday morning, at nine o'clock, was called to see a negro man aged twenty-six years. Found him mostly delirious; pulse one hundred and thirty—very full, hard and bounding; no cough; respiration very rapid. Suspected, from the physical signs present, pneumonia of the right lung. Tongue dry—yellow in the middle, with red point and edges; head drawn back; all the dorsal muscles rigid—the body could not be bent. He was bled, eight ounces of blood being abstracted. The pulse, after the bleeding, became even faster than before. The blood presented a peculiar appearance, the buffy coat marked. A large dose of calomel was given, and five drops tincture veratrum every three hours ordered. The next day, Sunday morning, the pulse was frequent, though soft; pupils widely dilated; eyes constantly open and staring; respiration sixty a minute; petechiæ on the chest; purpura on the legs—spots one-eighth of an inch in diameter; these were very indistinct to-day (Monday.)

The patient was attacked with a severe chill on Wednesday, followed by complete unconsciousness. He received no treatment till Saturday, and died Sunday at two o'clock. An autopsy was made by Drs. Raushenberg, W. F. Westmoreland, Connally, and J. T. Johnson. The brain was full of blood; the arachnoid and pia mater were intensely congested; the dura mater was cut just below the optic commissures, and a milky fluid oozed out. In attempting to separate the arachnoid from the pia mater, several adhesions were found. An inflammatory adhesion, two inches in length, was found on the left of the longitudinal fissure. The right lung was congested. The cerebellum and medulla oblongata were removed, and presented to the Academy by Dr. Raushenberg.

Dr. W. F. Westmoreland thought the adhesion on the top of the brain was not due to recent inflammation.

Dr. Cumming inquired if the inflammation extended down the spinal cord.

Dr. W. F. Westmoreland.—Yes; fluid poured out when the cord was cut as low down as it could be reached with the scalpel.

Dr. Armstrong.—Examined thoroughly the specimen presented by Dr. Raushenberg, and remarked: That effusions, as is well known to the members of the Academy, take place in the sub-arachnoid space. This space is filled by areolar tissue and blood-vessels. This exudation—for it is an *exudation*—being of a semi-solid or gelatinous consistence, is poured out from these vessels, and always covers, in the cases he had seen, one-third or one-half of the brain. Here is a case presenting the most marked injection he ever saw, and at the same time the most remarkable absence of exudation. The meninges of the cord seem to be even more inflamed than those of the brain.

Dr. Raushenberg.—Remarked that the membranes of the cerebrum contained a considerable amount of effused liquid.

Dr. Armstrong.—Has always found a considerable amount of effusion.

Dr. W. F. Westmoreland.—Thinks the state of the brain depends upon the length of the attack. The same changes would not be found when death occurred in a few hours as would be found in this case, continuing for three or four days.

Dr. J. G. Westmoreland inquired what killed, if death took place before inflammation was set up.

Dr. W. F. Westmoreland.—In many cases of cholera, death is not caused by exhaustion, produced by excessive discharges from the alimentary canal; but many persons fall on the floor, with no warning of its approach. Just so with cerebro-spinal meningitis—or influenza, as he prefers to designate it. Has seen persons attacked who were corpses in five hours. They were not killed by inflammation.

Dr. Bozeman.—Has seen patients die on the third or fifth day in typhoid fever, or the second or third day in scarlet fever. What causes death? Believes it to be due to the fact that the system is surcharged with their peculiar poisons.

Dr. J. G. Westmoreland.—What is found, post-mortem, in cases of meningitis of short duration?

Dr. W. F. Westmoreland.—Congestion, not sufficient to kill. Death due to shock caused by the rapid introduction of the peculiar poison into the system.

Dr. Armstrong.—Once made an autopsy of a man who walked home from his business, and retired as usual at night; was found unconscious, in bed, the next morning; died during the day of meningitis. Post-mortem made before night, and the same changes found as in cases of longer duration.

Dr. W. F. Westmoreland.—Have you ever made an autopsy of a person who died from shock?

Dr. Armstrong.—Yes, of cases with pulses not over sixty a minute.

Dr. W. F. Westmoreland.—That may be so, and shock not be present. Once examined a case attacked at eleven A.M.; died at five P.M. same day. Found no effusion and no inflammation.

Dr. Bozeman inquired why Dr. Westmoreland preferred the term malignant influenza to cerebro-spinal meningitis. He thought the latter an unusually appropriate name. The former expressed no pathological condition—neither did it indicate the seat of the lesion; it means nothing—simply a malignant *influence.*

Dr. W. F. Westmoreland.—Thinks this peculiar *influence* shows itself in different forms, producing various results. Does not always produce meningitis.

Dr. Stacy inquired what had been the most successful mode of treating this disease?

Dr. W. F. Westmoreland.—Treats with quinine, opium and calomel, with a blister to the spine. Is not satisfied with the treatment; only a small per cent. of his cases recover.

Dr. Cooper, of Americus, inquired if there was not, in this case, a remarkable absence of spasmodic muscular action. Thought the absence of this symptom was likely to mislead the physician. What length of time usually elapses before pus is formed?

Dr. Armstrong.—Has never seen it under ten days.

Dr. W. F. Westmoreland.—Had examined, *post-mortem*, only one case lasting over three or four days; that had continued for three or four weeks. The brain was completely disorganized.

Dr. Cooper related the case of a negro boy who died of meningitis fifty hours after he was attacked. The whole cerebrum was covered with a layer of free pus. Was surprised at the remarkably rapid formation.

The Doctor then called attention to the value of large doses of the tincture of chloride of iron in the treatment of hemorrhagic malarial fever. Quinine seemed to be of little use in this disease.

